# Simplified Analysis and Expanded Profiles of Avenanthramides in Oat Grains

**DOI:** 10.3390/foods11040560

**Published:** 2022-02-16

**Authors:** Mike Woolman, Keshun Liu

**Affiliations:** Grain Chemistry and Utilization Laboratory, National Small Grains and Potato Germplasm Research Unit, U.S. Department of Agriculture, Agricultural Research Service (USDA-ARS), 1691 S. 2700 W., Aberdeen, ID 83210, USA; mike.woolman@usda.gov

**Keywords:** oats, avenanthramides, HPLC method, analysis, extraction, AVA composition, grains

## Abstract

Uniquely, oats contain avenanthramides (AVAs), a group of phenolic alkaloids, exhibiting many health benefits. AVA analysis involves extraction with alcohol-based solvents and HPLC separation with UV and/or mass spectrometer detectors. There are many reported methods to extract AVAs. Almost all entail multiple extractions. The whole procedure is time- and labor-intensive. Furthermore, most quantifications are limited to three common AVAs (2f, 2p, 2c). The present study compared three extraction methods (all at 50 °C) for their effects on AVA concentrations and composition (% relative to total AVA) of oat grains. These included triplicate extractions with 80% ethanol containing 10 mM phosphate buffer (pH 2.0) (A), triplicate extractions with 80% ethanol (B), and a single extraction with 80% ethanol (C), while keeping solid/total solvent ratio at 1/60 (g/mL) and total extraction time of 60 min. Results showed that 80% buffered ethanol gave significantly lower AVA contents than 80% ethanol, while single and triplicate extractions with 80% ethanol produced the same extractability. However, the extraction method had no effect on AVA composition. Using 0.25 g sample size instead of 0.5 g saved extractants by half, without affecting AVA measurements. Consequently, a simplified method of extraction was developed, featuring Method C. The present study also expanded profiling individual AVAs beyond AVA 2c, 2p and 2f. Other AVAs identified and semi-quantified included 5p, 4p, 3f/4f, and 2pd. The simplified analysis was validated by measuring 16 selected oat grain samples. Some of these grains had relatively high contents of 4p, 3f/4f and 2pd, which have been considered minor AVAs previously.

## 1. Introduction

Compared to other cereal crops, oat (*Avena sativa* L.) is well known for its higher contents of oil, protein, and beta-glucan. Beta-glucan is a water-soluble dietary fiber and has been clinically shown to lower serum cholesterol levels in humans [1]. Furthermore, unique to oats is the natural presence of a group of phenolic alkaloids known as avenanthramides (AVAs) [2,3,4,5,6]. They were originally identified as phytoalexins produced by the plant in response to exposure to pathogens [7,8]. At the same time, AVAs were found to provide health benefits in mammals, including anti-oxidation, anti-inflammation, anti-atherosclerosis, and anti-cancer properties [9,10,11].

**Figure 1:**
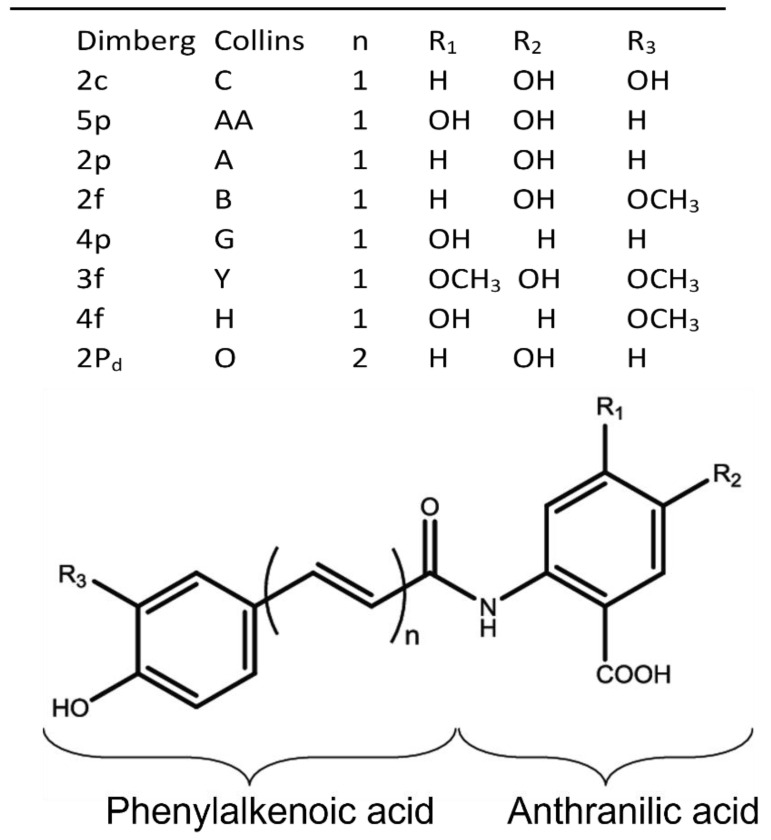
General structure of avenanthramides (AVAs), with two nomenclature systems proposed by Dimberg and Collins. AVAs shown in the mini table are those identified and based on the elution order from a common HPLC chromatogram. Adapted from Wise [5] and Liu and Wise [12].

Structurally, AVAs contain an anthranilic acid moiety conjugated to a phenylalkenoic acid moiety through an amide bond (Figure 1). There are two naming systems to properly describe individual AVAs. The Collins nomenclature is based on letters only [2], while the modified Dimberg system is based on a combination of a number denoting the type of anthranilic acid and a letter denoting the type of phenylalkenoic acid [4,5,6,11]. There are five major forms of the anthranilic acid moiety, denoting 1 for anthranilic acid, 2 for 5-hydroxyanthranilic acid, 3 for 5-hydroxy-4-methoxyanthranic acid, 4 for 4-hydroxyanthranic acid, and 5 for 4,5-dihydroxyanthranic acid. The phenylalkenoic acid moiety can be a derivative of hydroxycinnamic or avenalumic acid [3]. There are three main derivatives of hydroxycinnamic acid, including p for *p*-coumaric acid, f for ferulic acid, and c for caffeic acid, while the three corresponding avenalumic acid derivatives include the ethylenic homologues of the *p*-coumaric (denoted as pd), ferulic (fd), and caffeic (cd) acids [3,5]. In this paper, the number-letter system is used, as this system is becoming more popular. However, for easy reference, corresponding capital letters are also provided in parenthesis based on the Collins’ system.

AVAs are present in various tissues of oat plants [5]. Although as many as 35 AVAs have been identified [6], only AVA 2c, 2p, and 2f are commonly quantified due to a lack of purified forms as standards for other AVAs [13,14,15]. However, over the years, quite a few reports attempted to semi-quantify one or more “minor” AVAs, mostly based on retention time and existing standards of 2p, 2f and 2c. Examples included semi-quantification of AVA 5p [12], 5p and 4p [16], 5p, 4p, 3f/4f, and 2dp [17], 2fd and 2pd [18], 2s, 1c, 1p, 1f, and 1s [19], 2fd, 2pd, 3p, 3f, and 5f [20], and 8 [21], 10 [22] or 22 [23] total distinguishable AVA peaks (The last three all included the common three).

Analysis of AVAs typically involves extraction from oat tissues (leaves, roots, seeds, etc.) with an alcohol-based solvent and separation by liquid chromatography with ultra-violet [13,15,16,22] and/or mass spectrometer detection [6,21,23,24,25]. Regarding AVA extraction, there are many variations among reports, with respect to solvent type (methanol or ethanol solution at various concentrations, with or without a buffer), solid to solvent ratio, extraction time, temperature, and frequency, and the method to reduce extracts to dryness [6,13,15,16,18,21,22,24,25]. Almost all investigators make repeated extractions, which is time- and labor-intensive. In some cases, a rotary evaporator is used to dry extracts, which makes quantitative measurement difficult to achieve, due to use of a very small volume of an alcohol to reconstitute the dried extract. Although some researchers have tried to optimize their extraction procedures for maximum AVA contents [18,26], none has investigated the effect of AVA extraction on composition of AVAs (% relative to total) in oat samples.

In the authors’ USDA laboratory, a prior work dealt with the distribution of AVA 2p, 2f, 2c and 5p within oat kernels and its effects on the AVA contents of the remaining pearled kernels as the pearling cycle progressed [12]. The present study aimed to simplify AVA extraction procedure and provide expanded profiles of AVAs in whole oat grains. The effect of extraction procedures on contents and composition of AVAs in the same sample was also investigated.

## 2. Materials and Methods

### 2.1. Oat Grain Materials

Sixteen oat grain samples, compromising fourteen varieties with two originating from different sources, were collected from breeders in the North American Mountain and Midwest regions. Since the objective of the present study was not to investigate the growing environment effect, which has been completed by many previous investigators, no details were provided regarding their growing year and location. Rather, these samples were used to validate the performance of the simplified method developed in the present study for AVA analysis. Among the 16 samples, only one was hulless and the rest were hulled grains. All seed samples were winnowed with a lab winnower (Seedburo Equipment Co., Chicago, IL, USA) to remove debris. The cleaned grains were ground with a bladed coffee grinder to pass the U.S. No. 50 mesh (equivalent to 0.3 mm sieve openings).

### 2.2. Experimental Design for AVA Extraction

Several procedures of AVA extraction were compared, using a finely ground sample of oat variety, Ajay. Variations centered on (1) sample size (0.5 and 0.25 g), (2) extractant (80% ethanol containing 10 mM NaH_2_PO_4_ buffer (pH 2.0) or 80% ethanol alone), (3) frequency of extraction (1 or 3 times). When the sample mass was decreased from 0.5 to 0.25 g, the ratio of the sample mass (g) to the total solvent volume (mL) remained the same as 1/60. When the sample was re-extracted two more times (total three times), the total volume of the extractant (15 mL for 0.25 g or 30 mL for 0.5 g sample) and total extraction time (60 min) was kept the same by dividing them by three for each extraction. Also, when 80% buffered ethanol was used as an extractant, one time extraction was not conducted. Therefore, with each sample size (0.5 or 0.25 g), there were three extraction methods. Method A: three-time extraction with buffered ethanol; Method B: three-time extraction with 80% ethanol; and Method C: one-time extraction with 80% ethanol. There was a total of six combinations of Sample size x Extraction method).

Regardless of variations in sample size, extractant and extraction frequency as just described, a general procedure for all extractions is described herein. Weighed samples were extracted with a given volume of extractant in capped 50 mL plastic centrifuge tubes and magnetically stirred at 300 rpm for desired durations. All extractions took place at 50 °C, using a stainless-steel tray filled with water. A plastic rack with the centrifuge tubes was put in the tray, while the water in the tray was kept at 50 °C by a hotplate/stirrer with both temperature probe and speed controls (Isotemp, 7.5 in × 7.5 in plate size, Fisher Scientific, Waltham, MA, USA). After each extraction, an extract (supernatant) was obtained by centrifugation (1800× *g* for 10 min) and then transferred to a test tube having a cap. For the single extraction method (Method C), the extraction duration was 60 min, but for triplicate extractions (Methods A and B), extraction time for each extraction was 20 min (the total time was 60 min). The extracts were combined if repeated extractions were performed. A single extract or combined extracts were dried at 50 °C in a vacuum oven overnight. For redissolving a dried extract, 1 mL methanol was added to the tube containing the extract, then capped and vortexed. Redissolved extracts were filtered using microfuge tubes with a 0.2 µm nylon membrane and spinning at 9000 rpm for 1 min. After discarding the membrane, each filtrate was stored in a freezer in the capped and parafilm-sealed microfuge tube until HPLC analysis.

### 2.3. HPLC Analysis

HPLC analyses of all extracts obtained by Methods A, B and C in Section 2.2 were equally conducted, using a Waters HPLC system (Waters 600 Controller with Waters 600 pump, Waters Corp., Milford, MA, USA) coupled with a Waters 2487 Dual λ Absorbance Detector (a two-channel, tunable, ultraviolet/visible detector). The system utilized a Supelco reverse phase column (Discovery HS C18, 5µm diameter, 4.6 mm × 5 cm) with a pre-column filter and a guard column. The mobile phase consisted of solvent A (HPLC grade water containing 5% acetonitrile and 0.1% formic acid) and solvent B (acetonitrile with 0.1% formic acid). At a flow rate of 1 mL/min, the mobile phase gradient started with 100% solvent A and lasted for 10 min, followed by a linear gradient of solvent B from 13 to 30% (solvent A from 87 to 70%) over the next 12 min. The column was flushed with 100% of solvent B via gradient starting at 25 min for 5 min and then equilibrated back with 100% solvent A within 5 min. The column was maintained at 35 °C. All analyses were made with 10.0 μL injections. Signals were monitored at dual wavelengths of 280 and 330 nm. AVA 2p, 2f, and 2c were quantified by comparison of their peak areas at 330 nm with those of external standard curves developed using the corresponding authentic purified AVAs. The three standards, purified AVA 2p, 2f, and 2c, were kindly provided by Dr. Mitchell L. Wise, retired research plant biologist, USDA-ARS, Madison, Wisconsin, USA. AVA 5p, 4p, 3f, 4f, and 2pd were identified by their retention time [17] and mass spectral data as determined by liquid chromatography and mass spectrometry [16] and quantified using an average curve of the three AVA (2p, 2f, and 2c) standards. 

### 2.4. Validation of the Simplified Method with 16 Oat Grain Samples

Among the 6 combinations of sample size × extraction method described in Section 2.2, the one that consumed the least time, labor, and reagents, but at the same time gave a maximum total AVA content would be considered the simplified method. It was then used to extract 16 oat grain samples (described in Section 2.1) for AVA analysis. The objectives were (1) to validate the performance of the newly revised method for AVA analysis and (2) to show AVA profile of these samples beyond the three common AVAs of 2c, 2p, and 2f. 

### 2.5. Data Analysis

Duplicate AVA analyses starting from weighing samples were made for each of six procedures in extracting AVAs from the same Ajay oat grain. Each individual AVA was quantified for its concentration and composition (relative % to total AVA content) under each extraction procedure. Data were analyzed with Excel for calculating means and standard deviations. Three-way (sample size, extraction method, and AVA) analysis of variance (ANOVA) was conducted to determine the effect of each factor on individual and total AVA contents as well as AVA composition, using JMP software, version 12 (SAS Institute Inc., Cary, NC, USA). For the means of sample size × extraction method, the Tukey honestly significant difference (HSD) test was used for pair-wise comparisons at a significant level of *p* < 0.05. One way ANOVA was also conducted to determine the effect of oat sample (variety) on contents of individual and total AVAs. Pair-wise comparisons were also made by the Tukey HSD test at a significant level of *p* < 0.05.

## 3. Results and Discussion

The whole AVA extraction procedure involves four distinct Steps: (1) extracting a weighed amount of sample with a given volume of an extractant at a given temperature under mechanical or magnetic stirring; (2) centrifuging to recover extracts (supernatants), (with residues saved for repeated extractions if the procedure calls for); (3) reducing the volume (for concentrating AVAs) of an extract or combined extracts (if repeated extraction is carried out) by drying at a temperature similar to or lower than the extraction temperature, typically under a vacuum; and (4) redissolving the concentrated or dried extract with a given volume of methanol. There are great variations among reports, pertaining to steps (1) and (3) [6,13,14,15,16,18,19,21,22,24,25,26,27]. Variations in Step 1 center on sample size, extractant, the ratio of sample mass to extractant volume, the extraction temperature, duration, and the frequency (the initial extraction plus the number of repeated extractions). Regarding extractants, there are two major solvent systems: (1) ethanol or methanol at varying concentrations [6,13,14,19,22,26] and (2) 80% ethanol containing 10 mM phosphate buffer. The buffer pH is 2.8 [8,15,18] or 2.0 [16,17], before mixing with ethanol. In addition, 80% ethanol acidified with acetic acid to pH 2.0 was also used [21,27]. Regardless of the solvent type, almost all have duplicate or triplicate extractions (initial extraction plus 1 or 2 repeated extractions) for a single weighed sample. Variations in Step (3) include use of a rotary evaporator under a vacuum [15,16], a vacuum centrifuge evaporator [18], other means under a vacuum without mention of a specific equipment [13,14,21,22,25] or no mention of how extracts are dried [6].

To simplify AVA extraction, for Step 1, we first extracted 0.5 g of an oat grain sample (Ajay variety) by three methods: A, triplicate extraction with 80% buffered ethanol; B, triplicate extraction with 80% ethanol; and C, single extraction with 80% ethanol, while keeping the ratio of sample mass to total solvent volume the same (1 g/60 mL) for all three methods. To minimize extractant use, we also reduced sample size from 0.5 g to 0.25 g for another set of comparisons, while keeping the same ratio of sample mass to total solvent volume. For Step 3, although we had two rotatory evaporators, we found that using them to concentrate/dry extracts was time and labor consuming and difficult to transfer extracts quantitatively to test tubes after redissolving with 1 mL methanol. To solve these issues, we dried our extracts using a vacuum oven at 50 °C, although using a vacuum centrifuge evaporator could also be a good option. 

### 3.1. HPLC Chromatograms

**Figure 2:**
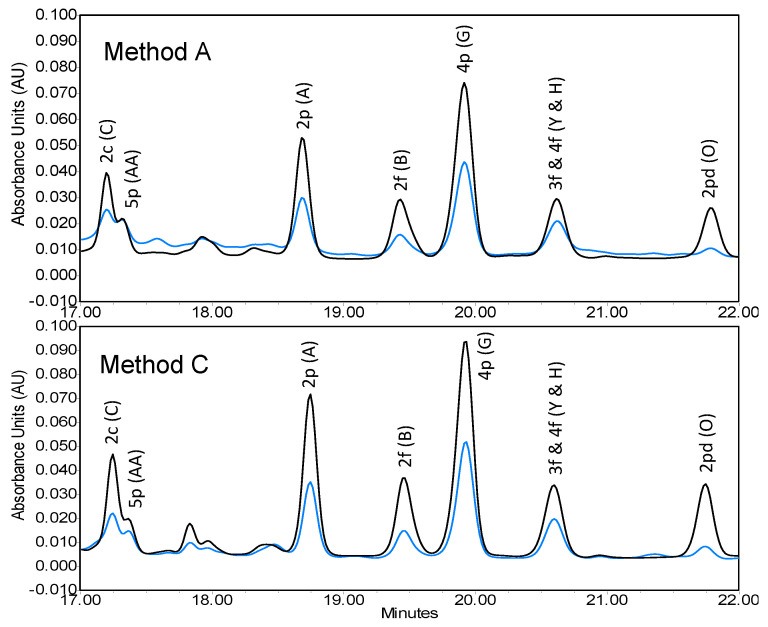
HPLC chromatogram of avenanthramides extracted from 0.5 g oat grain (Ajay variety) by Methods A and C. Absorbance units were monitored at dual wavelengths of 280 nm (blue) and 330 nm (black).

By analysis of extracts with the HPLC system described in Section 2.3, all three methods for 0.5 g samples showed similar chromatograms in terms of AVA separations (Figure 2). Since Methods B and C had identical chromatograms, only Method C was shown in Figure 2. Careful examination reveals that Method A showed some minor differences from the other two methods. These included (1) near 18 min retention time, Method A gave one unidentified peak while Methods B and C showed two peaks, (2) all peaks by Method A were smaller than those by the other two, and (3) Method A gave a slightly different signal response at 280 nm from those of Methods B and C. When the 0.25 g sample was extracted by the three methods, chromatograms were identical to these shown in Figure 1, except that the peak heights (areas) were reduced to near half (data not shown).

**Figure 3:**
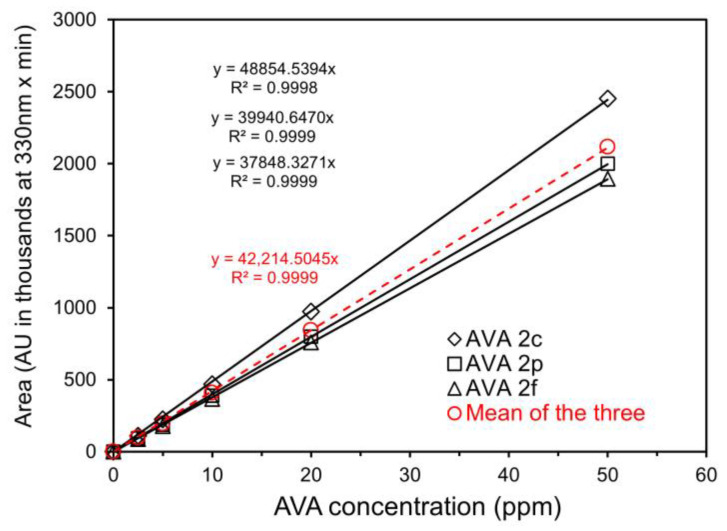
Standard curves connecting HPLC peak area (absorbance units in thousands at 330 nm × min) with concentrations (ppm) of the three avenanthramides (2c, 2p and 2f) and with their means. The order of the equations follows that in the legend.

Regardless of the minor differences among the three methods, the HPLC chromatograms of the oat grain sample obtained in the present study (Figure 2) were found to be close to that of the oat leaf sample obtained by Ren and Wise [17]), with respect to separation patterns of AVAs. There was a variation in AVA peak heights relative to each other between the two studies, apparently due to the differences in relative concentrations between the two types of tissues and/or varieties. Results also show that all extraction methods separated individual AVAs very well (Figure 2). For the present study, the three common AVAs, 2c, 2p and 2f, were identified and quantified by external standards of their purified forms, respectively, based on their corresponding retention time and peak areas at 330 nm. Identification of the other AVAs (5p, 4p, 3f, 4f, and 2pd) was based on retention time and HPLC-mass spectrometry conducted by the previous investigators [16,17]. Semi-quantification of these AVAs was made using the mean curve of the three AVAs (2c, 2p, and 2f) standards (Figure 3). This contrasted with use of 2f, 2p and 2c for semi-quantifying other AVAs having corresponding phenylalkenoic acid moiety (e.g., 2p for 5p, 4p or 2pd, and 2f for 3f and 4f) [16,17] or 2p alone for semi-quantifying all other AVAs [14,21,22]. Furthermore, being consistent with the findings of Ren and Wise [17], the chromatographic system used in the present study could not differentiate AVA 3f and 4f. Thus, for quantitative estimation, the two were combined and reported as a pair. In contrast to Wise [16] and Ren and Wise [17], AVA 2c and 5p were separated to such an extent that the two could be quantified individually by the Waters HPLC program.

### 3.2. Effect of Extraction Methods on AVA Concentrations and Compositions

When identified AVAs on HPLC chromatograms were quantified for each extraction method run in duplicate, the effect of extraction method on AVA concentrations and compositions of the same grain sample (Ajay variety) could be documented (Table 1). The concentrations included individual AVAs and total AVA, and expressed as µg/g sample (ppm, as is basis), while the AVA composition was expressed as a % relative to total AVA for each individual one. Upon three-way ANOVA, which covered three factors: extraction method (A, B and C), sample size (0.5 g and 0.25 g) and AVA (2c, 2p, 2f, 5p, 4p, 3f/f4, 2pd and total), it was found that the extraction method had significant effect on AVA contents (*p* < 0.05) (Table 2). Methods B and C gave the same AVA concentrations while Method A gave significantly lower AVA values than the other two methods. This finding was consistent with the peak height differences in HPLC chromatograms observed for the three methods (Figure 2).

Between Methods A and B, the only difference was that Method A used 80% buffered ethanol while Method B used 80% ethanol alone as the extractant. To find out possible reasons, we measured the pH of extractants and suspensions of extractant-oat grain sample. The pH of 80% ethanol was 7.4. Upon mixing with oat grain flour at 1/60 solid/solvent ratio (g/mL), it dropped to 6.4. When 10 mM phosphate buffer (pH 2.0) was added to 80% ethanol, the pH of the mixture was 4.3. Upon mixing with oat grain flour at the same solid/solvent ratio, the pH raised slightly to 4.5. The observed significantly less extraction of AVAs by Method A could result from the difference in pH between the two extractants used in the two methods. It could also result from the difference in ionic strength between the two extractants, as adding the phosphate buffer surely increased the ionic strength. 

As expected, the sample size had no effect, since the ratio of sample mass (g) to total solvent volume (mL) was kept the same (0.5/30 = 0.25/15 = 1/60). The interaction of Extraction method × Sample size had a significant effect, but the conclusion was the same as with individual factors (Table 1). Among the eight AVA attributes (individuals and total), there were great variations within each method. This finding was consistent with all previous reports which showed different concentrations of individual AVAs within the same samples and among samples [12,13,14]. 

Furthermore, the AVA composition shown in Table 1 and the ANOVA effect (Table 2) also indicated that both extraction method and sample size had no significant effect (*p* < 0.05) on AVA composition for the same grain sample. Therefore, although extraction method had a significant effect on AVA concentration, it had no effect on AVA composition. This finding is new and significant. So far, no previous studies have investigated the effect of extraction method on AVA composition.

The effect of extraction method on AVA content and method optimization have been carried out previously. Maliarova et al. [26] used a response surface methodology to optimize AVA extraction from oats. Their experimental design consisted of three factors: methanol concentration (60–100%), extraction temperature (20 to 80 °C), and extraction time (12–348 min), while their general procedure consisted of milling an oat grain to pass a 0.5 mm sieve, extracting 0.3 mg with a solvent at 1:10 solid to solvent ratio on an orbital thermo-shaker, in the dark, centrifuging to recover supernatants. The supernatants were not dried but stored. Later, they were cleaned by repeated centrifugations before injecting into HPLC directly without dilution. With this general procedure, they found that the optimal conditions for the highest yield of AVAs were a methanol concentration of 70%, extraction temperature at 55 °C, and time for 165 min. Pridal et al. [18] compared methods of extraction for maximum AVA extractability, with variations in extractants (80% buffered ethanol or 100% methanol), sample amount (0.1 or 1g), solid to solvent ratio (1:5 or 1:10), with or without sonication, extraction frequency (3, 4 or 5), and type of evaporation (rotary or vacuum centrifuge evaporator). All extractions were conducted at room temperature. When sonication was used for certain combinations, it was added between vortexing and mechanical mixing. With this general extraction procedure, they found that (1) 80% ethanol in phosphate buffer led to greater extraction than 100% methanol; (2) addition of sonication improved extraction; and (3) the use of a centrifuge vacuum evaporator leading to a threefold improvement in peak areas over the rotary evaporator. Their optimized extraction procedure featured triplicate extraction of 0.1 g sample with the 80% buffered ethanol and drying combined extracts with a centrifuge vacuum evaporator. For each extraction, 1:10 solid to solvent ratio is used while 10 min sonication was added between vortexing (after adding solvent to a sample) and 20 min mechanical mixing. 

Since the present study used extraction parameters (such as comparison of the 80% buffered ethanol with 80% ethanol, use of 1/60 solid to solvent ratio, and extraction at 50°C with magnetic mixing) different from those of Maliarova et al. [26] and Pridal et al. [18], results could not be directly compared among the three. Furthermore, the objective of the present study was to simplify AVA extraction without compromising AVA extractability. In contrast, the previous two studies focused on maximizing AVA extraction within the parameters chosen under each study. It is worthy to note that Pridal et al. [18] carried out their extractions at room temperature because they reasoned that using a water bath for heating and maintaining a higher temperature would impede the ability to have constant, thorough mixing of a sample during extraction. For the present study, we effectively solved the problem by using a hotplate/stirrer with both temperature probe and magnetic mixing speed controls. Using capped tubes in a water bath with a shaking device or a submersible magnetic mixer would also work.

### 3.3. Simplified Extraction Method

The present study showed that Method A (triplicate extractions with 80% buffered ethanol) extracted significantly less AVAs from oat grains than Methods B and C, while Method C (a single extraction with 80% ethanol) gave the same extractability as Method B (triplicate extractions with 80% ethanol). Therefore, Method C was selected as the simplified method for AVA extraction from oats. It consisted of a single extraction of oat samples (ground to pass a sieve having 0.3 mm openings) with 80% ethanol, with a solid (g) to solvent (mL) ratio = 1/60, at 50 °C with magnetic stirring for 60 min, centrifugation to recover an extract, drying of the extract at a low temperature under a vacuum, and re-solubilization of the dried extract with 1 mL of methanol. Although the three methods used the same total extraction time, since Method C required only one extraction and a single centrifugation to recover a single extract, it was less time- and labor- intensive compared to Methods A and B. Furthermore, when Method C is coupled with the use of a lower sample size (such as 0.25 g instead of 0.5 g) it could also reduce a significant amount of the extractant and drying effort, without affecting AVA contents and composition (Table 1).

Several groups of investigators have used 80% ethanol as an extractant for AVA extraction [13,14,22,24,25]. The extraction methods in these reports varied greatly in the solid to solvent ratio, extraction temperature and time, and the force (or a device) for mixing, but all required at least one repeated extraction after the initial extraction with 80% ethanol. Although the idea of having 80% ethanol as the sole extractant is not new, the present study was the first to show that with 80% ethanol as the extractant, a single extraction gave the same AVA extractability as triplicate extractions, when extraction was carried out at 50 °C and total extraction time was kept at 60 min. The same observation was made when the sample size was reduced to half and the solid to solvent ratio was kept at 1:60 (g/mL). The present study was also the first to show that variations in extraction methods did not significantly affect AVA composition in oat grains.

### 3.4. Individual and Total AVA Concentrations in 16 Oat Grain Samples

For verifying the performance of the simplified method of AVA extraction developed in the present study, as many as 16 oat grain samples were extracted for AVAs using Method C with the 0.25 g sample size described in Section 2.2 and Section 3.3. Three common AVAs and a few others were subsequently separated and quantified based on the HPLC analysis described in Section 2.3 and standard curves shown in Figure 3. Results indicate that the simplified analysis was sensitive and robust, providing individual AVA measurements with good repeatability (Table 3). Results also show that among the varieties there was a great variation in concentrations of individual AVAs and total AVAs. For the same variety (such as Morgan and CDC Dancer) AVA contents also varied with sources, as they were most likely grown in a different environments and crop years. Among the 16 grain samples, AVA 2c ranged from 2.19 to 42.81 μg/g; 5p from 0.51 to 5.17 μg/g; 2p from 3.28 to 36.88 μg/g; 2f from 2.96 to 38.16 μg/g; 4p from 1.17 to 42.59 μg/g; 3f and 4f from 1.63 to 15.00 μg/g; 2pd from 1.10 to 13.68 μg/g; and the total AVA from 15.88 μg/g in Wabasha to 144.14 μg/g in Maida. For most grain samples, 2c, 2p and 2f were indeed the most common ones with higher concentrations than other AVAs. However, in quite a few samples, 4p was present in substantial amounts. For some of them, such as Morgan 1, Ajay, Lamont (hulless), and Wabasha, 4p was close to or the highest among the AVAs measured. Some samples even had relatively high concentrations of 3f/4f and 2pd as well. In contrast, 5p was indeed a minor AVA for almost all samples, as reported previously [12].

There have been many reports on contents of individual AVAs and total AVA over the years. Bryngelsson et al. [13] reported that AVA 2c, 2p and 2f varied among seven Swedish oat varieties, with a total AVA content in groats ranging from 7.7 to 18.3 μg/g. Multari et al. [14] showed variations of AVA contents in eight oat cultivars, with 2c ranging from 3.5 to 39.2 μg/g; 2p, 6.8 to 29.6 μg/g; and 2f, 4.5 to 21.9 μg/g. Pridal et al. [18] reported that among six whole oat grains, total AVA (including 2f, 2p, 2c, 2pd and 2fd) ranged from 37 to 45 μg/g. Michels et al. [15] measured contents of the three main AVAs (2c, 2p and 2f) in 100 breeding lines and cultivars at three locations over two years. They found that for the top 10 performing genotypes, the total content of the three common AVAs (2f, 2p and 2f) ranged from 62.69 to 112.16 μg/g. Jagr et al. [6] showed that there are at least 35 AVAs present in the oat grain. Among them, AVA 2c, 2p, and 2f are the most abundant in oat grains, representing 65–70% of total AVAs. Bratt et al. [19] identified 2s, 1c, 1p, 1f and 1s (in addition to the three common AVAs) but found that these AVAs are not detected in groats and hulls. Hernandez-Hernandez et al. [22] quantified or semi-quantified 10 distinguishable AVA peaks and reported a total AVA range of 68.8 to 227.5 gμ/g in 17 oat lines and one variety. Dvorácek et al. [20] found that total AVA (2f, 2p, 2c, 2pd, 3p, 3f, 2fd, and 5f) ranged from 25.2 to 407.4 μg/g in 240 selected oat cultivars and lines. In contrast, Xochitl et al. [21] found relative higher concentrations in oat grains of a single variety, with AVA 2c, 2p and 2f being 257.3, 153.4, and 195.0 μg/g. The present study not only confirmed the varietal difference in AVA 2c, 2f and 2p contents reported previously, but also provided new information on the presence and varietal difference of AVA 5p, 4p, 3f/4f, and 2pd. The large variation in total AVAs among reports could be attributed to differences in oat variety, growing environment, the degree of grain processing, analytical method, and the number of individual AVAs quantified among studies.

## 4. Conclusions

The present study found that, when other factors were kept consistent, buffered (and acidified) ethanol extracted significantly lower amounts of AVAs from oats than 80% ethanol alone. When using 80% ethanol and keeping the ratio of solid to total solvent volume fixed at 1/60 (g/mL) and total extraction time fixed for 60 min, single extraction and triplicate extractions produced the same amounts of AVAs. However, extraction methods had no effect on AVA composition for the same sample. Consequently, a simple method of extraction (a single extraction with 80% ethanol, 1g/60mL solid to solvent ratio, at 50 °C for 60 min) was developed. Combining the simplified extraction method with smaller sample size (such as 0.25 g instead of 0.5 g) could save labor and reagents, without affecting the content and composition of AVAs in oat grains. The present study also expanded profiling individual AVAs beyond common AVAs 2c, 2p and 2f. The improved analysis was validated by measuring 16 selected oat grain samples. 

## Figures and Tables

**Table 1:** Individual and total contents of avenanthramides (AVAs) and AVA composition in Ajay oat grains as affected by extraction method and simple size ^1^.

Sample Size (g)	0.50	0.50	0.50	0.25	0.25	0.25
Extraction method	A	B	C	A	B	C
Extractant ^2^	80% buffered ethanol	80% ethanol	80% ethanol	80% buffered ethanol	80% ethanol	80% ethanol
Extraction frequency	3	3	1	3	3	1
			AVA concentration (μg/g)			
2c (C)	8.35 ± 0.27	10.89 ± 0.57	10.71 ± 0.02	7.94 ± 0.38	12.68 ± 1.26	12.02 ± 1.37
5p (AA)	3.62 ± 0.27	3.67 ± 0.13	2.96 ± 0.20	2.54 ± 0.06	3.55 ± 0.26	3.61 ± 0.08
2p (A)	19.64 ± 2.36	25.51 ± 0.73	26.1 ± 0.09	17.61 ± 0.69	27.59 ± 1.78	27.61 ± 1.59
2f (B)	12.06 ± 0.10	16.14 ± 0.23	16.08 ± 0.44	11.05 ± 0.29	16.89 ± 1.23	16.99 ± 1.14
4p (G)	32.43 ± 2.46	40.08 ± 0.31	36.65 ± 0.22	31.46 ± 3.64	43.17 ± 1.51	42.59 ± 2.85
3f & 4f (Y & H)	11.27 ± 1.11	14.05 ± 0.07	14.07 ± 0.16	9.99 ± 0.53	14.81 ± 0.44	15.00 ± 0.57
2pd (O)	8.95 ± 0.17	13.15 ± 0.35	13.3 ± 0.01	8.86 ± 1.98	14.19 ± 0.57	13.68 ± 0.64
Total	96.32 ± 6.74	123.49 ± 1.68	122.86 ± 0.42	89.43 ± 6.99	132.86 ± 6.53	131.49 ± 8.08
Method × Sample Size ^3^	24.08 ^c^	30.87 ^ab^	30.72 ^b^	22.36 ^c^	32.22 ^a^	32.87 ^ab^
			AVA Composition (% Relative to Total AVA)			
2c (C)	8.68 ± 0.32	8.82 ± 0.34	8.71 ± 0.01	8.89 ± 0.27	9.53 ± 0.48	9.12 ± 0.48
5p (AA)	3.75 ± 0.02	2.97 ± 0.16	2.41 ± 0.17	2.85 ± 0.16	2.68 ± 0.33	2.75 ± 0.23
2p (A)	20.36 ± 1.02	20.65 ± 0.31	21.24 ± 0	19.72 ± 0.77	20.76 ± 0.32	21 ± 0.08
2f (B)	12.55 ± 0.78	13.07 ± 0.36	13.09 ± 0.31	12.4 ± 1.29	12.7 ± 0.31	12.92 ± 0.07
4p (G)	33.67 ± 0.19	32.46 ± 0.19	32.27 ± 0.07	35.12 ± 1.32	32.5 ± 0.46	32.38 ± 0.18
3f & 4f (Y & H)	11.69 ± 0.34	11.37 ± 0.10	11.45 ± 0.17	11.18 ± 0.28	11.14 ± 0.21	11.42 ± 0.27
2pd (O)	9.31 ± 0.47	10.65 ± 0.14	10.82 ± 0.03	9.85 ± 1.44	10.68 ± 0.09	10.41 ± 0.15
Method × Sample size ^2^	24.99 ^a^	25.01 ^a^	25.05 ^a^	25.11 ^a^	25.08 ^a^	25.12 ^a^

^1^ Mean of duplicate AVA measurements ± standard deviation; ^2^ 80% buffered ethanol: a solution of 80% ethanol containing 10 mM phosphate buffer (which had a pH of 2.0); ^3^ Means of Method × Sample size having different superscript letters differed significantly at *p* < 0.05.

**Table 2:** Statistical parameters by analysis of variations for avenanthramide (AVA) concentration and compositions affected by extraction method and sample size.

		AVA Concentration			AVA Composition (% relative to total)		
Source	Degree of Freedom	Sum of Squares	F Ratio	Prob > F	Sum of Squares	F Ratio	Prob > F
Extraction method	2	1616.02	148.4072	<0.0001	1.46 × 10^−^^5^	0	1
Sample size	1	20.61	3.7853	0.0576	1.04 × 10^−^^6^	0	0.9982
AVA	7	113,210.26	2970.48	<0.0001	84,072.939	56,609.81	<0.0001
Extraction method × Sample size	2	84.16	7.7287	0.0012	2.08 × 10^−^^6^	0	1
Method × AVA	14	2224.08	29.1784	<0.0001	18.115	6.0988	<0.0001
Sample size × AVA	7	33.17	0.8702	0.5367	2.254	1.5177	0.184
Extraction method × Sample size × AVA	14	114.93	1.5078	0.1446	3.076	1.0355	0.4366

**Table 3:** Individual and total avenanthramides (AVAs) in 16 oat grain samples comprising 14 varieties ^1,2,3^.

Variety/Source ^4^	2c (C)	5p (AA)	2p (A)	2f (B)	4p (G)	3f & 4f (Y & H)	2pd (O)	Total
Ajay	12.02 ± 1.37 ^d^	3.61 ± 0.08 ^ab^	27.61 ± 1.59 ^c^	16.99 ± 1.14 ^c^	42.59 ± 2.85 ^a^	15.00 ± 0.57 ^a^	13.68 ± 0.64 ^a^	131.49 ± 8.08 ^a^
CDC Dancer 1	15.92 ± 0.52 ^c^	3.48 ± 0.74 ^b^	26.59 ± 1.12 ^c^	29.86 ± 1.95 ^b^	10.8 ± 0.46 ^d^	7.71 ± 0.47 ^c^	7.26 ± 0.62 ^c^	101.62 ± 5.88 ^b^
CDC Dancer 2	12.81 ± 0.25 ^d^	3.12 ± 0.23 ^bc^	18.64 ± 0.76 ^d^	15.35 ± 0.50 ^cd^	9.67 ± 0.89 ^de^	3.45 ± 0.08 ^e^	5.32 ± 0.07 ^d^	68.37 ± 1.13 ^d^
CDC Minstrel	24.12 ± 0.45 ^b^	2.84 ± 0.45 ^bcd^	32.13 ± 1.98 ^b^	35.51 ± 3.11 ^a^	14.78 ± 0.72 ^c^	9.26 ± 1.02 ^b^	12.4 ± 0.26 ^a^	131.04 ± 5.12 ^a^
Charisma	2.19 ± 0.13 ^g^	0.78 ± 0.12 ^e^	6.65 ± 0.12 ^fg^	7.15 ± 0.13 ^e^	5.34 ± 0.05 ^fgh^	2.07 ± 0.13 ^ef^	2.57 ± 0.15 ^ef^	26.75 ± 0.59 ^fgh^
Hayden	5.62 ± 0.29 ^f^	0.88 ± 0.09 ^e^	5.95 ± 0.08 ^fg^	5.07 ± 0.04 ^e^	4.44 ± 0 ^fghi^	1.63 ± 0.02 ^fg^	1.15 ± 0.01 ^g^	24.73 ± 0.23 ^gh^
Hi Fi	12.07 ± 1.26 ^d^	1.17 ± 0.14 ^e^	12.33 ± 0.61 ^e^	14.79 ± 0.39 ^cd^	2.81 ± 0.03 ^hi^	1.58 ± 0.02 ^fg^	2.25 ± 0.06 e ^fg^	47.01 ± 2.03 ^e^
Jerry	3.15 ± 0.72 ^fg^	0.51 ± 0.08 ^e^	5.02 ± 0.43 ^g^	2.96 ± 0.5 ^e^	5.92 ± 0.06 ^fgh^	1.72 ± 0.02 ^fg^	1.70 ± 0.09 ^fg^	20.99 ± 1.76 ^gh^
Lamont ^5^	8.54 ± 0.47 ^e^	1.79 ± 0.28 ^cde^	21.99 ± 0.54 ^d^	15.13 ± 0.22 ^cd^	20.06 ± 0.77 ^b^	7.94 ± 0.08 ^bc^	10.93 ± 0.56 ^b^	86.38 ± 2.77 ^c^
Leggett	8.27 ± 0.17 ^e^	1.15 ± 0.37 ^e^	9.68 ± 0.53 ^ef^	12.1 ± 0.66 ^d^	3.43 ± 0.11 ^ghi^	2.58 ± 0.30 ^ef^	3.06 ± 0.18 ^e^	40.27 ± 0.39 ^ef^
Maida	42.81 ± 0.57 ^a^	5.17 ± 1.26 ^a^	36.88 ± 2.34 ^a^	38.16 ± 1.72 ^a^	6.2 ± 0.19 ^fg^	5.23 ± 0.54 ^d^	9.69 ± 0.63 ^b^	144.14 ± 5.79 ^a^
Morgan 1	9.4 ± 1.17 ^e^	1.13 ± 0.14 ^e^	12.91 ± 0.58 ^e^	16.66 ± 0.95 ^c^	5.45 ± 0.12 ^fgh^	3.38 ± 0.31 ^e^	4.75 ± 0.10 ^d^	53.68 ± 3.36 ^e^
Morgan 2	2.68 ± 0.08 ^g^	0.53 ± 0.12 ^e^	4.83 ± 0.21 ^g^	4.84 ± 0.41 ^e^	7.19 ± 0.79 ^ef^	1.9 ± 0.25 ^fg^	2.6 ± 0.25 ^ef^	24.57 ± 2.09 ^gh^
Otana	8.38 ± 0.12 ^e^	1.29 ± 0.16 ^de^	6.65 ± 0.17 ^fg^	7.07 ± 0.06 ^e^	3.38 ± 0.07 ^ghi^	1.71 ± 0.08 ^fg^	1.59 ± 0.01 ^fg^	30.08 ± 0.20 ^fg^
Summit	3.78 ± 0.28 ^fg^	0.48 ± 0.01 ^e^	3.28 ± 0.13 ^g^	5.48 ± 0.15 ^e^	1.17 ± 0.07 ^i^	0.59 ± 0.02 ^g^	1.1 ± 0.01 ^g^	15.88 ± 0.16 ^h^
Wabasha	2.82 ± 0.21 ^g^	0.64 ± 0.03 ^e^	4.49 ± 0.04 ^g^	3.14 ± 0.08 ^e^	6.31 ± 0.55 ^fg^	1.66 ± 0.02 ^fg^	1.19 ± 0.03 ^g^	20.23 ± 0.68 ^gh^

^1^ Mean of duplicate measurements ± standard deviation (AVA concentration was expressed in µg/g sample, as is basis). ^2^ AVA’s were extracted by Method C with 0.25 g sample size (Table 1). ^3^ Column means having different superscript letters differed significantly at *p* < 0.05. ^4^ Two oat varieties, Morgan and CDC Dancer, were collected from two sources (1 and 2). ^5^ Lamont was a hulless variety. All others were hulled varieties.

## Data Availability

Due to restrictions, data only available upon request.
